# HHV-8 transmission via saliva to soothe blood-sucking arthropod bites

**DOI:** 10.1038/sj.bjc.6602086

**Published:** 2004-08-10

**Authors:** M Coluzzi, M L Calabrò, D Manno, L Chieco-Bianchi, T F Schulz, V Ascoli

**Affiliations:** 1 Dipartimento di Scienze di Sanità Pubblica, Università La Sapienza, Roma, Italy; 2 Dipartimento di Scienze Oncologiche e Chirurgiche, Università di Padova, Italy; 3 Dipartimento di Medicina Sperimentale e Patologia, Università La Sapienza, Roma, Italy; 4 Department of Virology, Hannover Medical School, Germany

**Sir,**

We refer to the letter by JM Wojcicki (2003), which is an interesting contribution towards attempts to elucidate risk factors for transmission of the human herpesvirus 8 or Kaposi's sarcoma-associated herpesvirus (HHV-8/KSHV). The author emphasises the prevalence of nonsexual intrafamilial horizontal transmission, the frequent seroconversion in childhood and the probable role of saliva, which in about 15–20% of infected individuals contains measurable HHV-8 loads. In a large mother–child transmission study in South Africa, very high viral copy numbers in maternal saliva have been found to be associated with transmission to the child (Dedicoat *et al*, 2004). Wojcicki lists a series of cultural and medicinal practices involving the exchange of saliva as documented in the Human Relations Area Files (HRAF, 1997) at Yale University. Using this database, she reports on traditional rituals of socioanthropological interest but does not highlight a common and global use of saliva, which is to soothe blood-sucking arthropod bites, the ancestral human behaviour that some of us have recently hypothesised to play a key role in HHV-8 transmission (Coluzzi *et al*, 2002). The important consideration here is that the local immune response to the arthropod bite, resulting in skin reactions like swelling and itching, which foster the application of HHV-8-containing saliva (by spitting, licking or sucking, or by smearing saliva over the arthropod bite site), could promote infection by the virus since HHV-8 is known to infect several cell types involved in inflammation, for example, macrophages, B cells and endothelial cells. An arthropod able to provoke a strong inflammatory response would thus have the greatest potential to promote HHV-8 transmission (‘promoter arthropod hypothesis’; Coluzzi *et al*, 2002). In support of this hypothesis, we have recently reported a suggestive evidence of a decrease in HHV-8 seroprevalence in age cohorts born in Sardinia during the larviciding campaigns aiming at the eradication of the malaria vector *Anopheles labranchiae* in the early 1950s (Coluzzi *et al*, 2003). Incidentally, *A*. *labranchiae*, like most malaria vectors well adapted to humans, may not be a typical promoter, but living in marshland habitat it would rather act as marker of an environment eventually producing promoter insects like *Ochlerotatus caspius*, *Coquillettidia richiardii* and *Aedes vexans*. Other potential promoter insects are, in the Mediterranean area, sand flies (Phlebotomus spp), biting midges (Culicoides spp and Leptoconops spp) and, perhaps more important, black flies (Simulium spp). Glossinids (tze-tze flies) and Tabanids (horse flies) could also be relevant in promoting HHV-8 transmission in Africa.

Whether any one of the human practices using saliva, as highlighted by JM Wojcicki, is involved in HHV-8 transmission remains a fascinating question. One may extend the hypothesis that application of saliva to wounds, or the use of saliva in preparing medications applied to wounds, is a route for HHV-8 transmission. We believe that future studies aimed at understanding the impact of living conditions, cultural or medicinal practices, on HHV-8 transmission should be designed such as to investigate this hypothesis by including appropriate questionnaires. Since HHV-8 infection in African children may be associated with hepatitis B virus (HBV) infection (Mayama *et al*, 1998), and given the reported link between insect (*Simulium buissoni*) bites and the rate of HBV infection in a holoendemic island of the Marquesas archipelago, French Polynesia (Chanteau *et al*, 1993), the promoter arthropod could have a much wider relevance.
